# Curvature‐Directed Elongation and Chiral Tilting Drive Asymmetric Tissue Patterning

**DOI:** 10.1002/advs.77940

**Published:** 2026-09-27

**Authors:** Liyao Xu, Frank Daniel Peters, Haokang Zhang, Madison Stiefbold, Thangam Ramar, Aditya Agarwal, Leo Q. Wan

**Affiliations:** ^1^ Department of Biomedical Engineering Rensselaer Polytechnic Institute Troy New York USA; ^2^ Department of Biological Sciences Rensselaer Polytechnic Institute Troy New York USA; ^3^ Center For Biotechnology and Interdisciplinary Sciences Rensselaer Polytechnic Institute Troy New York USA; ^4^ Center For Modeling Simulation and Imaging in Medicine Rensselaer Polytechnic Institute Troy New York USA

**Keywords:** cell chirality, curvature sensing, symmetry breaking

## Abstract

Substrate topography, such as curvature, has been recognized as a key physical cue that regulates cell organization and tissue function, yet how curvature influences chiral cell behavior remains unclear. Here, we created curved surfaces (concave or convex) separated by flat regions to investigate the role of cell chirality in curvature‐guided multicellular morphogenesis. C2C12 cells showed opposite alignment biases on concave and convex surfaces, and the cells on flat regions adopted a reversed bias compared to those on the adjacent curved areas. Cells with opposite intrinsic or pharmacologically altered chirality exhibited correspondingly reversed alignment patterns. Detailed analyses revealed that local curvature initially directed cell elongation along distinct geometric axes on convex and concave surfaces, followed by biased tilting and migration governed by intrinsic cellular chirality. Together, our findings demonstrate that cell chirality superimposes a directional bias upon curvature‐guided elongation, thereby driving collective alignment and migration. This work provides new insights into how cells respond to curvature and highlights a potential mechanism for tissue‐level symmetry breaking, regulated by substrate topography.

## Graphical abstract

Curvature and cell handedness together break tissue symmetry. On convex and concave surfaces, cells elongate along opposite geometric axes and tilt in a consistent chirality‐driven direction, yielding opposite alignment patterns from the same cells. Reversing intrinsic cell chirality reverses both. This elongation–tilting mechanism links substrate topography to tissue left–right asymmetry in development and disease.

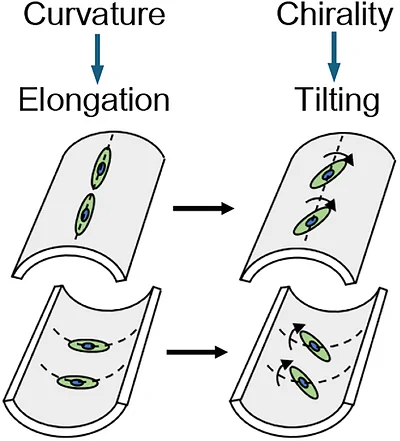

## 1 Introduction

Geometric constraints of the cell are widely recognized to influence cell morphology, motility, and function [1]. Among these constraints, substrate curvature, a primary descriptor of the large‐scale shape of three‐dimensional (3D) environments, has been studied in recent years due to its role in regulating cell morphology, alignment, migration, and multicellular organization [1, 2, 3, 4]. Numerous investigations have examined how tubular tissue structures can guide cell alignment, aligning cells either axially or circumferentially, depending on cell phenotype and whether the curvature is concave or convex [5, 6, 7, 8, 9, 10]. Interestingly, the cells also exhibit a consistent angular bias relative to the primary axis, driven by the intrinsic cell handedness or cell chirality, resulting in a helical alignment of the cellular tube, as observed in both engineered and native microvessels [11]. The underlying biophysical mechanisms of curvature‐directed chiral morphogenesis, however, have not been fully explored and remain elusive.

Over the past several decades, numerous studies have shown that cells respond to substrate curvature and exhibit preferential alignment, with cylindrical surfaces widely used as a model system. Many cell types, including chick heart fibroblasts [5], bone marrow stromal cells [8], human umbilical vein endothelial cells [9], and mouse embryonic fibroblasts [10], preferentially align along the axial direction on convex surfaces. In an early study, Dunn and Heath proposed that this curvature‐dependent alignment may arise because stress fibers resist bending, favoring the direction of least substrate curvature [5]. Biton and Safran, and later Bade et al., further developed this framework by considering the role of cellular contractility in regulating cell orientation on curved surfaces [10, 12]. However, these mechanisms primarily explain how cells respond to convex curvature and choose between axial and circumferential alignment, but do not account for left–right asymmetry. More recently, several detailed observations have reported chiral behavior of the cells on curved substrates. Jin et al. reported biased alignment of airway smooth muscle cells on convex surfaces [13]. Similarly, Ehrig and Schamberger observed left‐handed alignment of mouse pre‐osteoblasts on negatively curved surfaces after long‐term culture [14, 15]. In addition, we recently showed that endothelial cells in microvessel models adopt helical orientations within lumen structures, and that their handedness is determined by intrinsic cell chirality [11]. Here, we investigate cellular responses to substrate curvature from the perspective of cell chirality, focusing on the origin of left–right asymmetric alignment on curved surfaces.

Cell chirality is an intrinsic asymmetry at the cellular level and has been studied through a variety of approaches [16]. For example, at the multicellular level, multicellular tissue on ring‐ [17], strip‐ [18], or rectangle‐ [19] shaped micropatterns revealed collective chiral patterns. In addition, the chiral alignment of skeletal myotubes guided by microgrooves has also been reported [20]. In 3D, Chin et al. observed the chiral rotation of cell spheroids within a bilayer Matrigel system [21]. At the single‐cell level, when constrained to circular, elliptical, or T‐shaped adhesive islands, actin organization, nuclear rotation, and organelle positioning within cells exhibited asymmetry [19, 22, 23, 24]. Collectively, these studies suggest that cells can develop pronounced left–right asymmetries under geometric constraints. Yet it remains unclear how the tissue forms a chiral structure through the interplay between substrate curvature and intrinsic cellular chirality.

In this study, we employed substrates containing semi‐cylindrical structures with concave or convex curvature to investigate how curvature influences the emergence of chiral cellular alignment. We found that cells exhibit distinct alignment patterns in both curved regions and adjacent planar areas. Our results reveal that curvature provides an unbiased geometric cue that orients cell elongation, upon which intrinsic chirality superimposes a directional bias that determines the final cellular orientation. This discovery offers new insights into the interplay between curvature and chirality and suggests a novel mechanism for the emergence of tissue‐level asymmetry.

## 2 Results

### 2.1 Cellular Biased Alignment Depends on Surface Curvature

**FIGURE 1:**
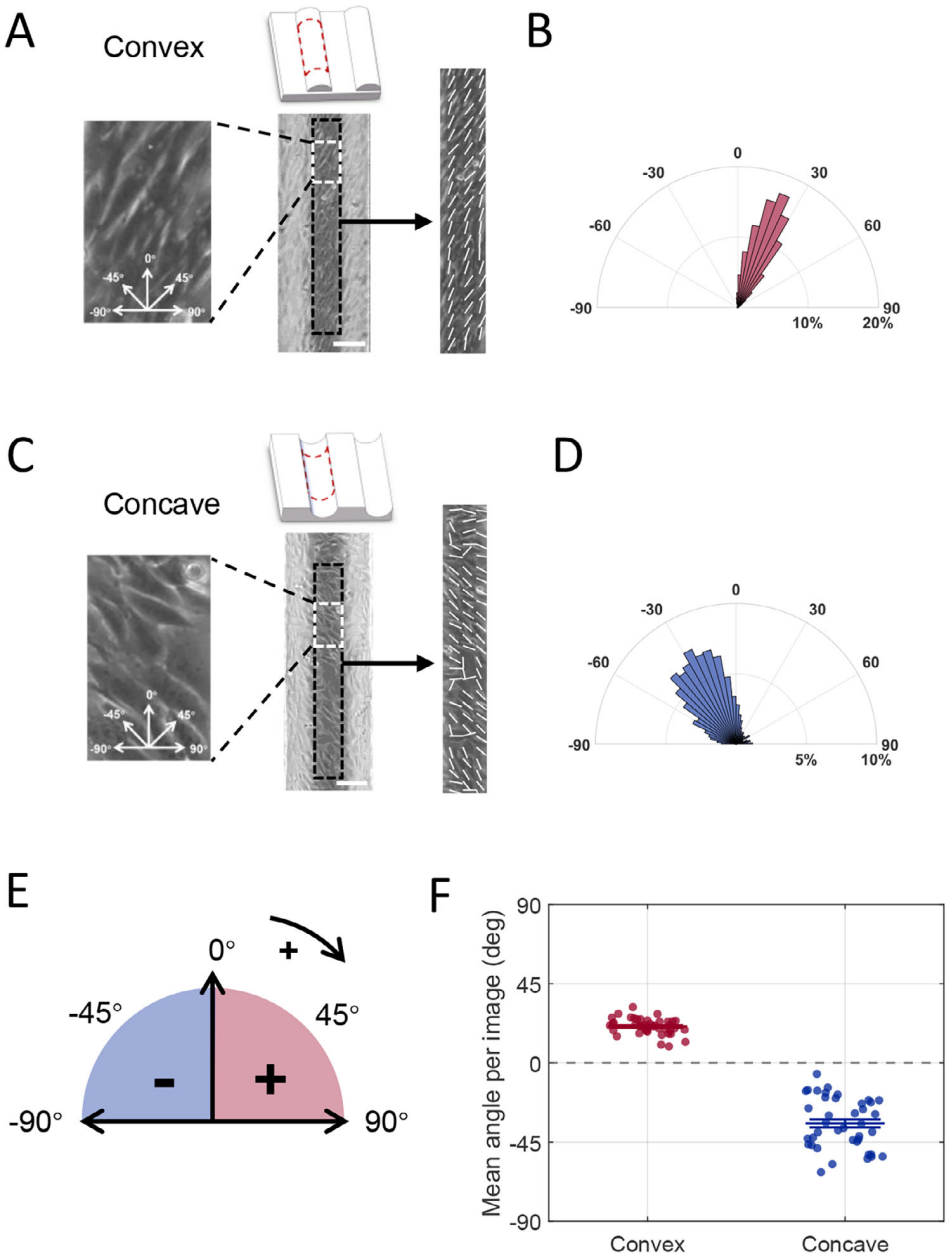
Curvature determines the directional bias of cell alignment. (A, C) C2C12 cells after 24‐h culture show a distinct difference in biased cell alignment on convex (A) and concave (C) surfaces, with zoomed‐in images on the left and images overlaid with local cell alignment (shown as white lines) on the right side. Scale bar = 100 µm. (B, D) Rose plots showing the distributions of local cell orientation angles on convex (B) and concave (D) surfaces. (E) Definition of cell orientation angles, where 0° represents cell alignment along the longitudinal axis of the cylindrical surface, while both + 90° and – 90° correspond to the circumferential direction. Clockwise deviation is defined as positive. (F) Mean orientation angles of cells on convex and concave surfaces. Each data point represents the mean axial orientation angle calculated from one cropped image. Error bars represent the standard error of the mean (SEM). n = 43 images for convex and n = 40 images for concave surfaces.

PDMS platforms featuring concave or convex curved surfaces were fabricated using a combination of stereolithography (SLA) 3D‐printed molds, spin coating, and PDMS double‐casting techniques. Hemicylindrical surfaces with a radius of curvature of approximately 150 µm were arranged at regular intervals and separated by planar regions approximately 300 µm in width. The mouse myoblast cell line C2C12 was seeded onto these platforms and cultured to form a confluent monolayer. Twenty‐four hours after cell seeding, C2C12 cells displayed strikingly different alignment orientations on convex and concave surfaces (Figure 1A–D). When the longitudinal axis was defined as 0°, the circumferential direction as ±90°, and clockwise deviation as positive (Figure 1E), positive and negative angles simply reflected the directional bias of cell alignment with respect to the longitudinal axis. Orientation analysis revealed that cells on convex surfaces preferentially aligned at positive angles, whereas cells on concave surfaces exhibited an opposite, negatively biased alignment (Figure 1B,D). The mean orientation angle was 20.74 ± 0.68° on convex surfaces and −34.45 ± 2.28° on concave surfaces (Figure 1F).

### 2.2 Cell Alignment Bias is Associated With Intrinsic Cell Chirality

**FIGURE 2:**
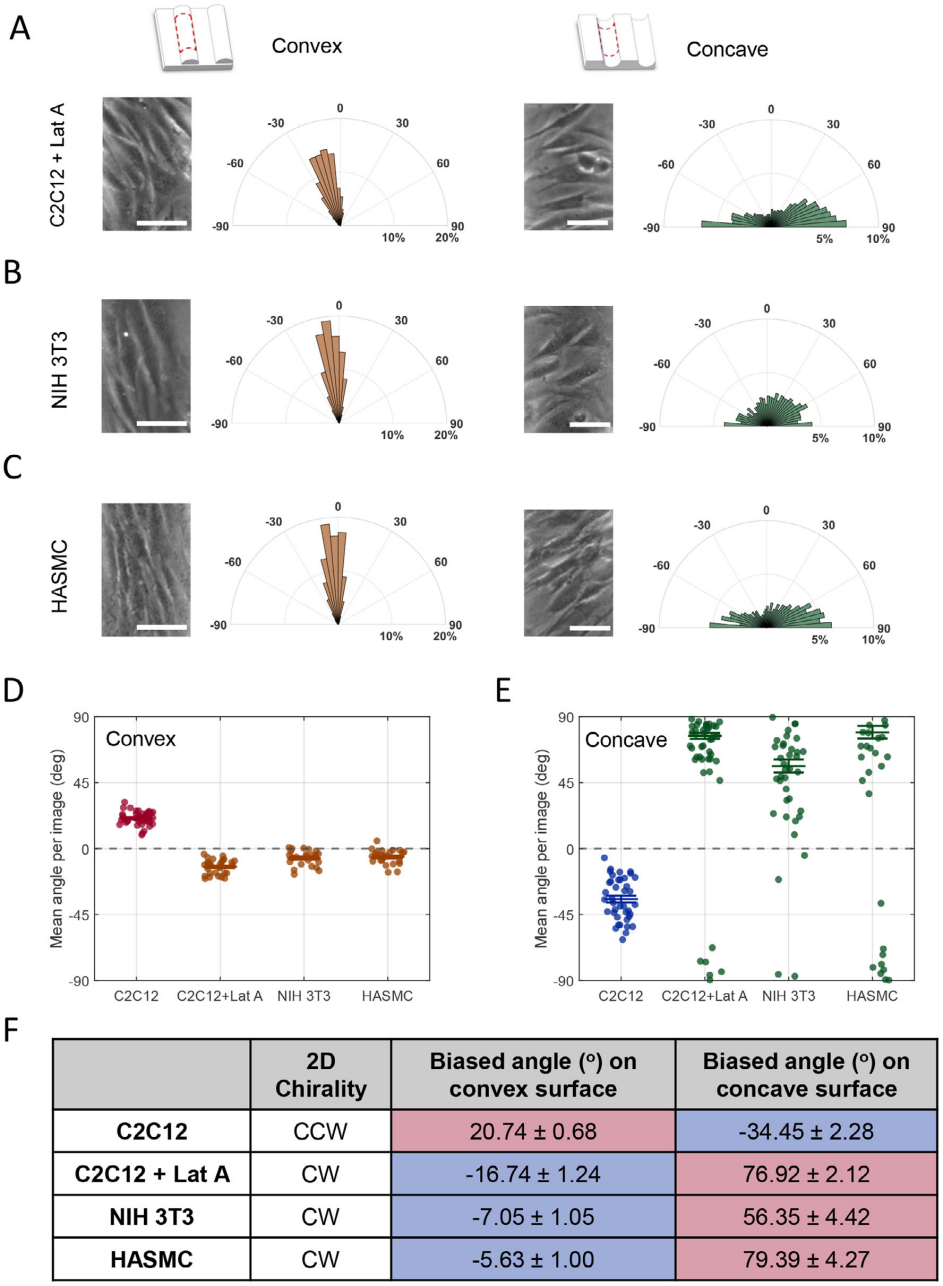
Cell alignment bias on topographical surfaces is associated with intrinsic cell chirality. (A) Representative phase‐contrast images and corresponding rose plots of orientation‐angle distributions for C2C12 cells treated with 50 nM Latrunculin A (Lat A) on convex and concave surfaces. (B) Representative phase‐contrast images and corresponding rose plots of orientation‐angle distributions for NIH 3T3 cells on convex and concave surfaces. (C) Representative phase‐contrast images and corresponding rose plots of orientation‐angle distributions for human aortic smooth muscle cells (HASMCs) on convex and concave surfaces. Scale bars = 50 µm. (D, E) Mean orientation angles of cells on convex (D) and concave (E) surfaces across different cell types and treatment conditions. Each point represents one cropped image; data are shown as mean ± SEM. n = 43, 30, 23, and 24 images for C2C12, C2C12 + Lat A, NIH 3T3, and HASMCs, respectively, on convex surfaces; n = 40, 44, 37, and 26 images, respectively, on concave surfaces. (F) Table of the chirality measured on two‐dimensional (2D) ring‐shaped micropatterns and mean orientation angles (mean ± SEM) determined on convex and concave surfaces. Chirality on 2D micropatterns was defined as clockwise (CW) or counterclockwise (CCW) according to [17].

To examine the origin of asymmetric alignment, we first sought to reverse the intrinsic chirality of C2C12 cells. Latrunculin A (Lat A), an inhibitor of actin polymerization, has been shown in multiple 2D studies to reverse C2C12 chirality at low concentrations [17, 24]. Notably, Lat A treatment reversed the direction of C2C12 alignment on curved surfaces, such that cells exhibited negatively biased orientations on convex regions and positively biased orientations on concave regions (Figure 2A,D,E).

Cell chirality has been shown to be an intrinsic, phenotype‐dependent property. Previous two‐dimensional studies have demonstrated that NIH 3T3 cells exhibit clockwise (CW) chirality on ring‐shaped micropatterns, whereas C2C12 cells exhibit counterclockwise (CCW) chirality [17]. Here, we further examined primary human aortic smooth muscle cells (HASMCs) and found that they also displayed CW chirality on 2D micropatterns (Figure S1). Both cell types exhibited chiral alignment opposite to that of C2C12 cells on curved surfaces, characterized by negatively biased orientations on convex regions and positively biased orientations on concave regions (Figure 2B–E). Figure 2F summarizes the intrinsic chirality of all groups on 2D micropatterns and their corresponding alignment angles on surfaces with different curvatures. Overall, cell chirality, as characterized on 2D ring‐shaped micropatterns, was consistent with the directional bias of cell alignment on curved surfaces: cells showing a CCW bias on 2D rings exhibited positive alignment angles on convex surfaces and negative angles on concave surfaces, whereas cells showing a CW bias exhibited the opposite trend on curved surfaces.

Cell contractility has been implicated in regulating both curvature‐dependent cell orientation [10, 12] and chiral behaviors across single‐cell and collective systems [18, 23, 25]. We, therefore, next examined whether modulating contractility with small‐molecule drugs alters cellular alignment bias on curved substrates. Cellular contractility was reduced by the ROCK inhibitor Y‐27632 or the myosin II inhibitor blebbistatin and enhanced by the Rho activator II (CN03). Cell orientation was analyzed 24 h after seeding for all treatment conditions. Despite noticeable changes in cell morphology, none of these treatments reversed the alignment bias on either convex or concave surfaces (Figure S2). However, the magnitude of the final alignment angle was altered under some conditions. On concave surfaces, Y‐27632‐ and blebbistatin‐treated cells exhibited alignment angles closer to the longitudinal direction, whereas CN03 treatment produced no significant difference. On convex surfaces, Y‐27632 and blebbistatin did not significantly alter the alignment angle, whereas CN03 treatment slightly reduced the magnitude of the alignment bias. These results indicate that contractility can modulate the magnitude of the alignment bias but cannot reverse its direction.

### 2.3 Distinct Roles of Curvature and Chirality in Establishing Biased Cellular Alignment

**FIGURE 3:**
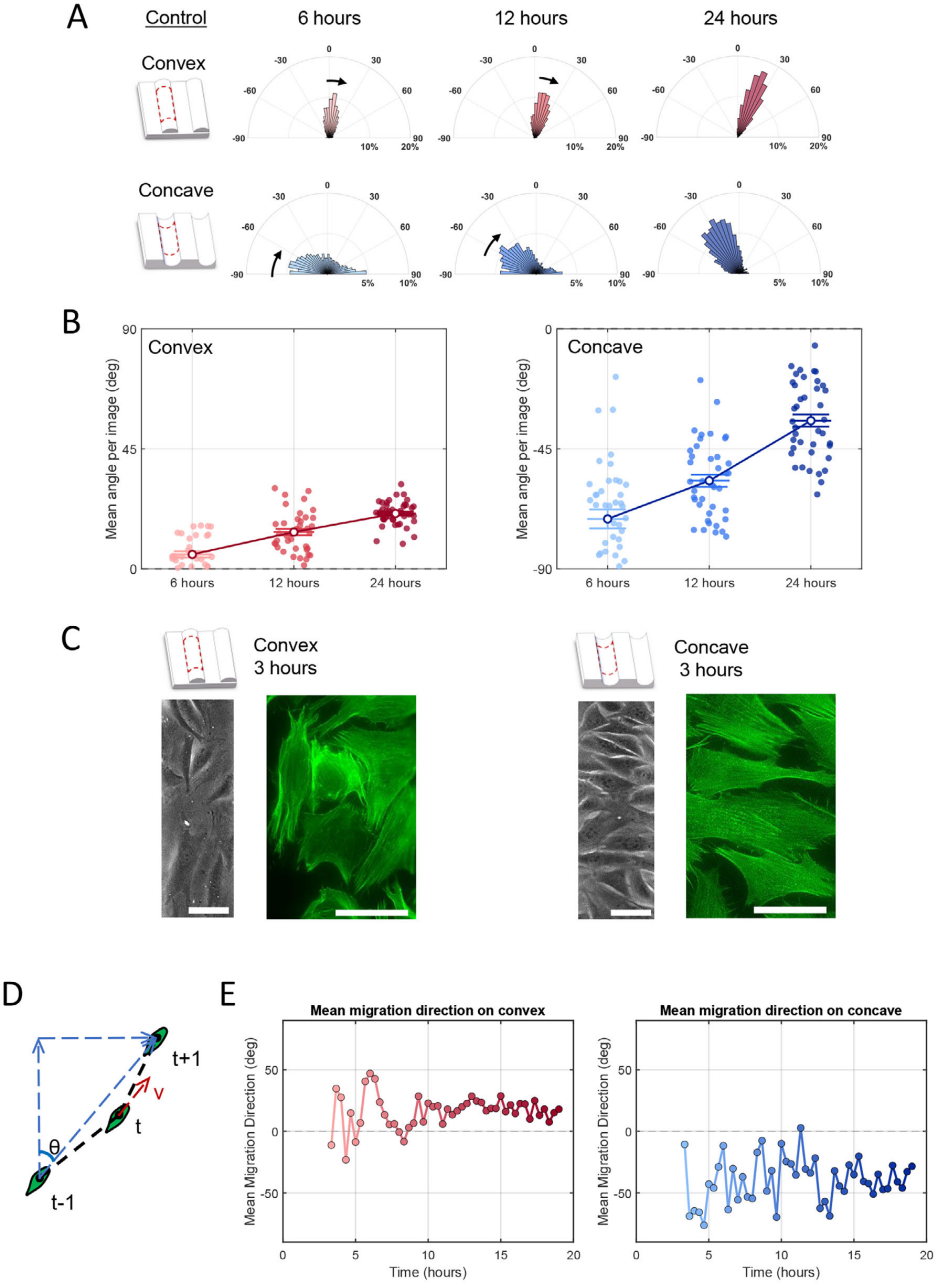
Geometric curvature sets the initial cell orientation, while cell chirality introduces a directional bias. (A) Rose plots showing the distributions of orientation angles of control C2C12 cells on convex and concave surfaces at 6, 12, and 24 h after seeding. (B) Mean orientation angles of control C2C12 cells on convex and concave surfaces at 6, 12, and 24 h after seeding. Each dot represents one image; data are shown as mean ± SEM. n = 33, 40, and 43 images for the 6, 12, and 24 h time points, respectively, on convex surfaces, and n = 44, 40, and 40 images, respectively, on concave surfaces. (C) Representative phase‐contrast images and fluorescence images of F‐actin in C2C12 cells on convex and concave surfaces at 3 h after seeding. F‐actin was labeled with Alexa Fluor 488‐conjugated phalloidin. Scale bars = 50 µm. (D) Definition of cell migration direction, where *t* denotes the current time frame, and *θ* represents the migration angle. (E) Temporal evolution of the mean migration direction of control C2C12 cells on convex (red, left) and concave (blue, right) surfaces.

To further explore the biophysical mechanism underlying the distinct biases in cell alignment on convex versus concave surfaces, we acquired phase‐contrast images at 6, 12, and 24 h after seeding and quantified cell orientation at each time point (Figure 3A,B). At early stages, cells exhibited markedly different orientations depending on surface curvature. Cells on convex surfaces were predominantly elongated along the longitudinal axis, whereas cells on concave surfaces were primarily aligned in the circumferential direction. Earlier time‐point images further showed that, as early as 3 h after seeding, both cell elongation and stress‐fiber organization already exhibited distinct curvature‐dependent preferences (Figure 3C). Over time, cells collectively deviated from their initial elongation directions in a clockwise fashion (as shown by arrows in Figure 3A), leading to positive alignment angles on convex surfaces and negative angles on concave surfaces at 24 h.

Similar trends were observed in Lat A‐treated C2C12 cells (Figure S3) and NIH 3T3 cells (Figure S4). In both cases, cells initially aligned along the longitudinal axis on convex surfaces and along the circumferential direction on concave surfaces. During culture, however, cells progressively tilted in a counterclockwise direction, ultimately resulting in alignment biases opposite to those of untreated C2C12 cells. One exception was observed for Lat A‐treated C2C12 cells on concave surfaces, where the alignment at 24 h appeared to return slightly toward the circumferential direction. Together, these observations support distinct roles for curvature and chirality, with curvature associated with the preferred elongation direction and chirality with the tilting bias.

Time‐lapse imaging further confirmed the progressive reorientation of cells over 24 h. On convex surfaces, C2C12 cells gradually deviated from the longitudinal axis to establish a positive alignment bias (Video S1), whereas on concave surfaces, cells initiated from a circumferential orientation and progressively deviated clockwise to establish a negative alignment bias (Video S2). To further examine cell migration during this process, cell nuclei were labeled with Hoechst 33342 and tracked over time (Videos S3 and S4), and migration directions were estimated from displacements between the preceding and following time points (Figure 3D). Although migration directions fluctuated substantially at early time points, a more consistent directional bias was observed at later stages of culture. Cells overall migrated toward positive angles on convex surfaces and negative angles on concave surfaces, consistent with the corresponding alignment biases (Figure 3E).

### 2.4 Curvature‐Dependent Alignment Bias Emerges at the Single‐Cell Level

To determine whether chiral alignment on curved surfaces depends on cell–cell interactions or collective behavior, C2C12 cells were sparsely seeded on convex and concave surfaces, and the orientation of individual cells was examined (Figure S5). At 6 h after seeding, single C2C12 cells already exhibited biased alignment on curved surfaces, with positive angles on convex surfaces and negative angles on concave surfaces. Lat A‐treated C2C12 cells exhibited the opposite alignment bias. These directional preferences were consistent with those observed in multicellular cultures, and stress fibers were aligned with the direction of cell elongation.

To examine whether cell seeding density affects multicellular chiral behavior, we compared C2C12 cultures seeded at 40, 80, and 120 k cells/cm^2^ and analyzed their alignment at 24 h (Figure S6). In all cases, the overall alignment biases were comparable. On convex surfaces, a slightly stronger alignment bias was observed at the highest seeding density compared with the lower‐density conditions, whereas no significant difference in alignment angle was observed among the different seeding densities on concave surfaces. Together, these results indicate that curvature‐dependent chiral alignment can emerge at the single‐cell level and does not require an established multicellular collective, while cell density may modulate the magnitude of the resulting alignment bias.

### 2.5 Cells in Adjacent Planar Regions Adopt a Biased Alignment Opposite to That of Neighboring Curved Surfaces

**FIGURE 4:**
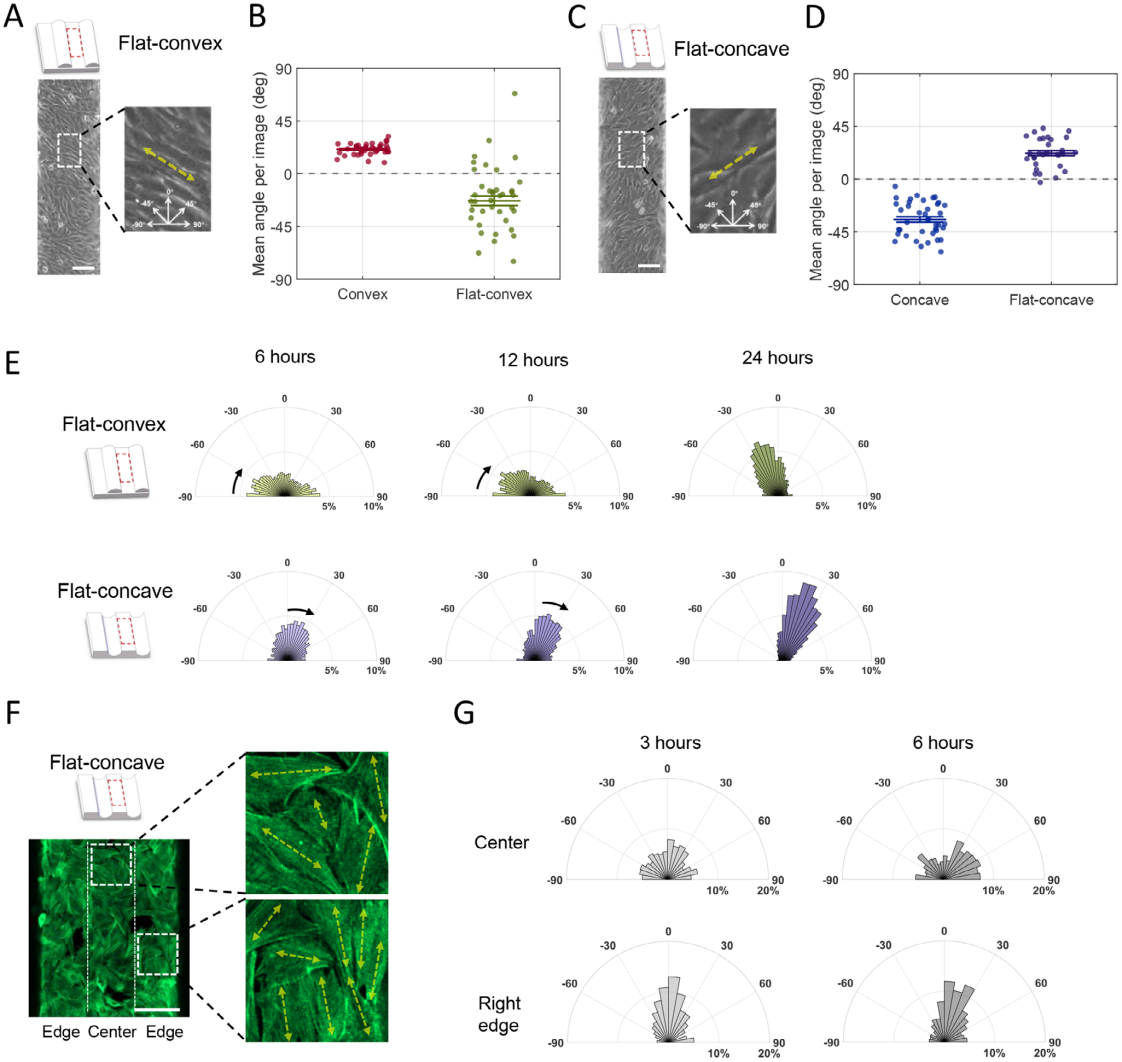
Cells in adjacent planar regions adopt a biased alignment opposite to neighboring curved surfaces. (A, C) Phase‐contrast images of C2C12 cells in planar regions adjacent to convex (Flat–convex; A) and concave (Flat–concave; C) surfaces 24 h after seeding. Scale bars = 100 µm. (B, D) Mean orientation angles of C2C12 cells on convex and Flat–convex regions (B) and on concave and Flat–concave regions (D). Each dot represents one image; data are shown as mean ± SEM. n = 43 and 39 images for convex and Flat–convex regions, respectively, and n = 40 and 32 images for concave and Flat–concave regions, respectively. (E) Rose plots showing the distributions of cell orientation angles in Flat–convex and Flat–concave regions at 6, 12, and 24 h after seeding. (F) Fluorescence images of C2C12 cells on Flat–concave regions 3 h after seeding. F‐actin was labeled with phalloidin. Magnified views on the right show cells located at the center (top) and edge (bottom) of the planar region. Scale bars = 100 µm. (G) Rose plots showing the distributions of cell orientation angles in the center and right‐edge subregions of Flat–concave areas at 3 and 6 h after seeding.

In planar or flat regions (approximately 300 µm in width) adjacent to each curved surface, cells also exhibited chiral alignment (Figure 4A,C). Planar regions neighboring convex surfaces were designated as Flat–convex, whereas those adjacent to concave surfaces were termed Flat‐concave. Cells in Flat–convex regions displayed negatively biased orientations, opposite to the chiral alignment observed on the neighboring convex surfaces (Figure 4B). Similarly, cells in Flat–concave regions exhibited positively biased orientations, again opposite to the alignment on adjacent concave surfaces (Figure 4D). As a result, cells across the entire PDMS substrate adopted a wave‐like global alignment pattern.

Orientation analysis at 6, 12, and 24 h, together with time‐lapse imaging, revealed similar temporal trends in these planar regions (Figure 4E and Videos S5 and S6). In the early stages, cells in Flat–convex regions primarily aligned horizontally, whereas cells in Flat–concave regions preferentially aligned vertically. Over the course of 24 h, cells in both planar regions progressively tilted clockwise, resulting in alignment biases opposite to those on the neighboring curved surfaces. In the additional groups examined—Lat A–treated C2C12 cells, NIH 3T3 cells, and HASMCs—the planar regions exhibited similar alignment behaviors: cells consistently aligned opposite to those on the neighboring curved surfaces (Figure S7).

To further examine the origin of asymmetric alignment in planar regions, we analyzed spatiotemporal cell orientation with actin fluorescence imaging at earlier stages: 3 and 6 h after seeding. Planar regions were subdivided into left edge, center, and right edge for orientation analysis (Figure 4F). As expected, in Flat–concave regions at 3 h, cells in the central area exhibited largely random orientations, whereas cells at the edges aligned preferentially along the Flat–concave boundary. By 6 h, cells displayed a clear tendency toward chiral alignment (Figure 4G). In Flat–convex regions, spatial differences between the edge and center were also observed, although cells in the central region already showed a predominantly horizontal orientation at 3 h, while a clearer directional bias was present near the boundaries (Figure S8). Together, these observations suggest that cells respond locally to the curved‐to‐flat transition regions, from which a consistent alignment bias may develop across the planar area.

## 3 Discussion/Conclusion

In this study, we find that cells may exhibit seemingly opposite asymmetric alignment and migration depending on environmental curvature. By pharmacologically reversing cell chirality and examining cell types with opposite intrinsic chirality, we demonstrated that the direction of bias on 3D curved substrates is associated with the chirality measured on 2D micropatterns. Time‐lapse imaging and orientation analyses across different time points further revealed that curvature primarily determines the initial elongation direction of cells, upon which intrinsic chirality imposes a directional tilting that ultimately establishes biased alignment. Moreover, the influence of curvature is not limited to the cells directly sensing the curved surface; local geometric cues at the curved‐to‐flat transition can propagate into neighboring planar regions, inducing asymmetric organization in surrounding cells.

**FIGURE 5:**
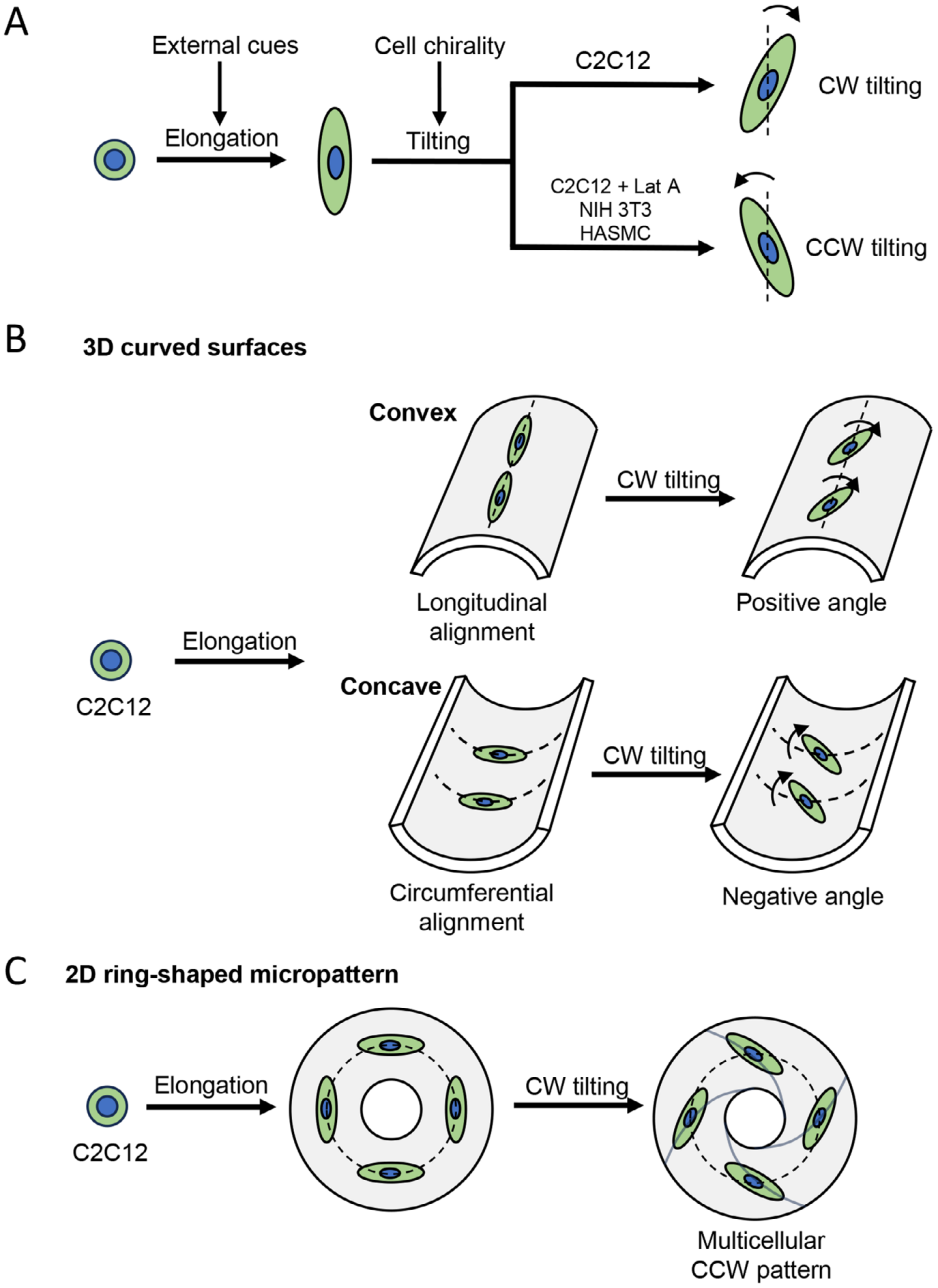
Schematic illustration of the elongation–tilting framework for chiral cell alignment. (A) Proposed elongation–tilting framework in which external cues such as topographical features and 2D geometry determine the primary direction of cell elongation, while intrinsic cell chirality dictates the direction of tilting. The interplay of the two factors gives rise to a directional bias in cell alignment. Different cell types or treatment conditions can exhibit different cell chirality, leading to either clockwise (CW) or counterclockwise (CCW) tilting. (B) Schematic illustration of how the elongation–tilting framework applies to C2C12 cells on topographical surfaces. On convex surfaces, curvature promotes longitudinal elongation, with CW tilting producing a positive orientation bias. On concave surfaces, curvature promotes circumferential elongation, with CW tilting producing a negative orientation bias. (C) Schematic illustration of how the same framework applies to C2C12 cells under geometric confinement on a two‐dimensional ring‐shaped micropattern. Cell elongation follows the local geometry of the micropattern, while CW tilting of cells gives rise to a collective CCW multicellular pattern.

Many studies have demonstrated that 3D curvature guides cell alignment and migration. However, despite extensive documentation of how various cell types respond to convex, concave, or more complex Gaussian curvatures, the underlying biomechanical mechanisms remain poorly understood. Rather than addressing the origin of this initial curvature‐dependent alignment, our study focuses on how intrinsic cell chirality contributes to asymmetric alignment and morphogenesis on curved surfaces. We propose an “elongation–tilting” framework in which external geometric cues define the preferred elongation axis, while intrinsic cell chirality biases the direction of tilting relative to that axis (Figure 5A). Because curvature establishes different preferred elongation axes on convex and concave surfaces, the same clockwise tilting of C2C12 cells produces positive and negative alignment biases, respectively (Figure 5B). In contrast, Lat A–treated C2C12 cells and NIH 3T3 cells retained similar curvature‐dependent elongation preferences but tilted counterclockwise, producing reversed alignment biases and further validating this framework.

Importantly, our results suggest that cell chirality is likely a major driver of the direction of tilting. This process, however, can be influenced by other factors. For example, Lat A‐treated C2C12 cells showed the expected reversal of tilting direction at early time points on both convex and concave surfaces (Figure S3). On concave surfaces, however, this reversed bias was relatively weak, with cell alignment almost shifting back toward the circumferential direction. In addition to reversing cell chirality, Lat A may alter actin polymerization‐dependent processes, including stress‐fiber remodeling and focal‐adhesion dynamics [26], thereby weakening or counterbalancing the expression of reversed chirality under the specific cytoskeletal and mechanical conditions of the concave surface. Although Y‐27632, CN03, and blebbistatin did not change the preferred curvature‐dependent elongation or the direction of chiral tilting, these perturbations altered the magnitude of the final alignment angle under some conditions. These results suggest that ROCK/myosin‐II‐dependent contractility is not a primary determinant of the direction of elongation or chiral tilting in our system but can modulate the extent of the resulting asymmetric alignment. Further mechanical modeling may help clarify how curvature‐guided elongation, chirality‐dependent tilting, and cell contractility together shape asymmetric alignment.

The “elongation–tilting” mechanism proposed here not only explains how cells respond to curvature but also provides a unifying framework for interpreting previous two‐dimensional studies (Figure 5C). For example, the ring‐shaped micropattern assay commonly used to assess cell chirality is consistent with this mechanism. Previous studies have shown that ring‐shaped geometry guides cells to align predominantly along the circumferential direction [27], corresponding to the preferred elongation axis in our framework. Owing to the intrinsic chirality of C2C12 cells, this tangential alignment then undergoes a clockwise deviation, leading to the emergence of a counterclockwise (CCW) multicellular pattern around the ring.

The proposed tilting mechanism is likely associated with biased actin organization, as observed in actin swirling within single‐cell patterns [19]. When single HFFs were confined to elongated elliptical islands, the actin cytoskeleton developed a directional tilt relative to the major axis. Upon release from the micropattern confinement, newly formed protrusions and changes in cell shape preferentially followed the tilted direction, providing a functional link between chiral actin organization and subsequent cell motile behavior [28]. This behavior closely parallels the cellular tilting observed in our system, and the observation that individual cells already exhibit the same biased orientation on curved surfaces suggests that multicellular chiral alignment originates from cell‐intrinsic chirality (Figure S5). Future work is necessary to identify how the actin cytoskeleton drives whole‐cell tilting and to determine the underlying molecular mechanisms.

The boundaries between the curved and flat regions appear to provide sufficient geometric cues for local cell chiral alignment (Figure 4F,G). Considering that the alignment bias in the flat region is opposite to that on the adjacent curved surface, we believe that this chiral alignment is likely influenced by the local curvature at the curved‐to‐flat transition, where the curvature sign is opposite to that of the neighboring cylindrical surface. This result is rather interesting, as it indicates that the cells not only sense cylindrical surfaces but also respond to the curvature formed between cylindrical surfaces and nearby flat regions. Previous work has also highlighted the role of curved‐to‐planar transition regions in regulating local cellular organization [29]. Future studies will be needed to determine the spatial range of this boundary effect and how competing cues from neighboring convex and concave regions influence planar cell organization.

Previous studies have demonstrated that many developmental asymmetry events are driven by cell chirality, such as cardiac looping in vertebrates [30] and the rotation of the hindgut epithelial tube in Drosophila [31, 32]. More recently, endothelial cells have also been shown to exhibit chiral alignment, which is associated with vascular permeability [11]. Notably, all examples occur in environments characterized by pronounced curvature, and it is important to investigate whether and how curvature may be an essential condition for determining or stabilizing asymmetric tissue development. Our findings emphasize that multicellular morphogenesis is jointly shaped by tissue curvature and intrinsic chirality, offering new perspectives on the mechanisms underlying such asymmetric phenomena.

## 4 Experimental Section

### 4.1 Platform Fabrication

The fabrication process was modified based on previous studies [33, 34]. To fabricate the platform containing both curved surfaces and flat regions, a combination of 3D printing and PDMS molding was employed. The 3D structure of the platform was designed using SolidWorks, and the mold was fabricated using a stereolithography (SLA)‐based 3D printer (Formlabs Form 2) with a resin material. PDMS (Sylgard 184) was prepared by mixing the silicone base and curing agent at a 10:1 weight ratio, poured into the 3D‐printed mold, and cured in an oven at 60°C for at least 3 h. Due to the surface roughness of the 3D‐printed mold and the limited precision in printing curved geometries, the meniscus of uncured PDMS was utilized to form a smooth concave surface. Specifically, a small volume of uncured PDMS was dropped onto the cured PDMS surface and spin‐coated at 3000 rpm for 3 min. The sample was then baked at 60°C for at least 2 h to obtain a smooth concave morphology. To fabricate the corresponding convex structure, a modified double‐casting method was adopted. The concave PDMS mold was treated with oxygen plasma for 30 s, followed by immersion in 100% ethanol for 30 min. After drying in an oven at 60°C for 30 min, the concave PDMS was used as a secondary mold for casting the convex PDMS counterpart.

### 4.2 Cell Culture, Seeding, and Treatment Procedures

The mouse myoblast cell line C2C12 and the fibroblast cell line NIH 3T3 were cultured in Dulbecco's Modified Eagle Medium (DMEM) supplemented with 10% fetal bovine serum (FBS), 1% penicillin‐streptomycin, and 1% sodium pyruvate. Human aortic smooth muscle cells (HASMCs; Primary Aortic Smooth Muscle Cells; Normal, Human, ATCC PCS‐100‐012) were obtained from the American Type Culture Collection (ATCC). Cells were cultured using the Vascular Smooth Muscle Cell Growth Kit (ATCC PCS‐100‐042) according to the manufacturer's protocol. PDMS substrates were cut into appropriate sizes to fit either 12‐well or 6‐well plates. The PDMS substrates were then sterilized using UV‐ozone treatment for 15 min. Following sterilization, the substrates were coated with fibronectin (Sigma‐Aldrich, F1141) by incubating them in a 50 µg/mL fibronectin solution prepared in phosphate‐buffered saline (PBS) for 30 min. After incubation, the PDMS surfaces were rinsed once with PBS and then immersed in medium for cell seeding. Cells were seeded onto curved PDMS surfaces at a density of approximately 80 k cells/cm^2^. After the addition of the cell suspension, the plates were gently agitated to ensure uniform distribution, and cells were allowed to settle and adhere to the substrate for 15 min before the medium was gently refreshed. For drug treatment, Latrunculin A (Sigma‐Aldrich, L5163), Y‐27632 (STEMCELL Technologies, 72302), blebbistatin (Calbiochem, 203390), and CN03 (Cytoskeleton, Inc., CN03‐A) were added to the refreshed medium after cell attachment. The working concentrations were 50 nM for Latrunculin A, 2–10 µM for Y‐27632, 2 µM for blebbistatin, and 2 µg/mL for CN03. Cells were incubated for 3, 6, 12, or 24 h prior to imaging.

### 4.3 Imaging and Fluorescence Staining

Phase‐contrast imaging was performed using a Keyence BZ‐X700 microscope. For phase‐contrast time‐lapse imaging, images were acquired every 12 min and continued for 24 h. For fluorescence imaging, stained samples were imaged using either a Zeiss LSM 800 confocal microscope or a Keyence BZ‐X700 microscope. Following incubation for the designated time periods, cells were gently rinsed with PBS and fixed with 4% paraformaldehyde for 15 min at room temperature. The samples were then washed three times with PBS (5 min each). Subsequently, cells were permeabilized with 0.1% Triton X‐100 for 10 min and then blocked with 1% bovine serum albumin (BSA) for 30 min to reduce nonspecific binding. Actin filaments were stained using Alexa Fluor 488‐conjugated phalloidin (Aat Bioquest, 23115) diluted 1:300 in blocking buffer and incubated for 30 min. After staining, the samples were washed three times with PBS, each for 5 min. After washing, the samples were stored in PBS until imaging.

### 4.4 Cell Alignment Analysis

Cell orientation was quantified using a gradient‐based analysis method based on the previous work [17]. All image processing and analysis were performed in MATLAB. Specifically, captured images were first rotated and cropped to align the cylindrical axis vertically. Nearest‐neighbor interpolation was applied during rotation to minimize transformation artifacts. The processed images were then divided into subregions of defined size (typically 40–50 pixels with 0.75 µm/pixel). For each subregion, the principal orientation was computed based on the image gradient. Because cell orientation represents an axial direction without distinction between the two ends of the cell axis, orientation angles (θ_*i*_) were defined from −90° to 90° with a periodicity of 180°. Mean orientation angles were therefore calculated using double‐angle circular statistics rather than a simple arithmetic mean, such that orientations near +90° and −90° were treated as neighboring axial orientations. The mean angle $\bar{\theta }$ was calculated by $\bar{\theta }=\frac{1}{2}\;atan2\left(S,C\right)$, where *atan*2 stands for the four‐quadrant inverse tangent function, *C* $=\frac{1}{n}\sum_{i=1}^{n}\cos\left(2\theta_{i}\right)$, and $S=\frac{1}{n}\sum_{i=1}^{n}\sin\left(2\theta_{i}\right)$. For each image, the axial circular mean across all subregion orientations was calculated to obtain the mean alignment angle for that image. At the group level, the axial circular mean of all per‐image mean angles within the same experimental group was calculated to obtain the overall group mean angle.

### 4.5 Nucleus Tracking Analysis

Time‐lapse imaging was performed using a Keyence microscope. To enable nucleus tracking, the medium was refreshed with Hoechst 33342 (working concentration: 1 µM) 15 min after cell attachment. Importantly, cells were stained for only 6 min to minimize phototoxicity, after which the medium was refreshed again. Time‐lapse imaging was initiated 3 h post‐seeding, with images acquired every 20 min. Exposure time was minimized to further reduce phototoxic effects. The acquired image sequences were imported into the u‐track MATLAB package [35], in which nucleus tracking was performed using appropriate tracking parameters. The output included the coordinates of each nucleus trajectory. Custom MATLAB scripts were developed to visualize trajectories and compute cell velocities. A sliding window approach (window size = 3 frames) was used to calculate instantaneous velocity at each time point. The mean migration direction per frame was computed using circular statistics based on the velocity vectors of all tracked cells.

### 4.6 Single Cell Alignment Experiment

For single‐cell alignment analysis, C2C12 cells were sparsely seeded onto PDMS substrates at a density of 3 × 10^4^ cells per well in 12‐well plates (i.e., 8 k cells/cm^2^) to maintain separation between individual cells. After 6 h of culture, cells were fixed and stained for actin filaments with phalloidin following the same procedure described above. Based on the organization of the actin cytoskeleton, the long axis of each individual cell was defined as the cell elongation direction. A line segment was manually drawn along the long axis of each cell in ImageJ, and its orientation angle was recorded using the ROI Manager. The measured angles were subsequently imported into MATLAB for visualization.

### 4.7 2D Micropatterning Assay

Micropatterned substrates were fabricated using established microcontact patterning approaches as previously described [36]. Briefly, microscale geometric features were defined on gold‐coated glass slides using PDMS stamps replicated from a silicon master mold fabricated by SU‐8 photolithography. Adhesive self‐assembled monolayers (SAMs) of octadecanethiol were transferred to the gold surface to define cell‐adhesive regions, while non‐adhesive ethylene glycol‐terminated SAMs were used to prevent cell adhesion in the background. Patterned substrates were coated with fibronectin (50 µg/mL) prior to cell seeding. Cells cultured on ring‐shaped micropatterns were imaged 24 h after seeding using phase‐contrast microscopy (10×). Cell alignment on ring patterns was quantified using a custom MATLAB‐based analysis, in which local orientation was determined using an intensity gradient‐based approach applied to 32 × 32 pixel (24 × 24 µm) regions, as described previously [17, 36].

### 4.8 Statistical Analysis

Orientation data were obtained as described in the Cell Alignment Analysis section. Briefly, axial orientation angles were first determined for individual image subregions and summarized within each cropped image to obtain a mean axial angle for that image. No additional normalization or outlier exclusion was performed before statistical analysis. Rose plots were used to show the distribution of subregion‐level orientation angles, whereas swarm plots and group‐level statistical analyses were based on the mean axial angle of each cropped image. Group data are presented as the circular mean ± SEM calculated from the image mean angles. The sample size (n) denotes the number of images, which were randomly selected for imaging and analysis. Differences in mean orientation between groups were assessed using the Watson–Williams test applied to doubled angles. For experiments involving multiple pairwise comparisons, *p* values were adjusted using the Holm method to control the family‐wise error rate. Statistical significance was defined as *p* < 0.05. All statistical analyses were performed in MATLAB.

For 2D micropattern experiments, data were derived from independent individual ring patterns. The frequencies of clockwise (CW) and counterclockwise (CCW) alignment were compared using a two‐tailed binomial test under the null hypothesis of equal probability (*p* = 0.5).

## Author Contributions

Conceptualization: L.X. and L.Q.W.; Methodology: L.X., F.D.P., H.Z., M.S., T.R., and L.Q.W.; Investigation: L.X., F.D.P., and A.A.; Formal analysis: L.X.; Writing – original draft: L.X.; Writing – review and editing: All authors. Supervision: L.Q.W.; Funding acquisition: L.Q.W.

## Use of Generative AI and AI‐Assisted Technologies in the Writing Process

AI‐assisted tools were used for language editing, including grammar correction and sentence restructuring to improve clarity and readability. All AI‐assisted edits were reviewed and verified by the authors, who take full responsibility for the content of the manuscript.

## Conflicts of Interest

The authors declare no conflicts of interest.

## Supporting information




**Supporting File 1**: advs77940‐sup‐0001‐VideoS1.avi.

**Supporting File 2**: advs77940‐sup‐0002‐VideoS2.avi.

**Supporting File 3**: advs77940‐sup‐0003‐VideoS3.avi.

**Supporting File 4**: advs77940‐sup‐0004‐VideoS4.avi.

**Supporting File 5**: advs77940‐sup‐0005‐VideoS5.avi.

**Supporting File 6**: advs77940‐sup‐0006‐VideoS6.avi.

**Supporting File 7**: advs77940‐sup‐0007‐SuppMat.pdf.

## Data Availability

The data that support the findings of this study are available within the article and its Supporting Information. Additional data are available from the corresponding author upon reasonable request.
