# Supplementary material for: Curvature‐Directed Elongation and Chiral Tilting Drive Asymmetric Tissue Patterning
Source: Adv Sci (Weinh). 2026 Sep 27:e77940. Online ahead of print. doi: 10.1002/advs.77940 (PMC13616418; doi:10.1002/advs.77940)
Supplement: Supplementary file 1 [file ADVS-9999-e77940-s001.pdf]

Supporting Information

Curvature-Directed Elongation and Chiral Tilting Drive Asymmetric Tissue Patterning

Liyao Xu[1], Frank Daniel Peters[1], Haokang Zhang[1], Madison Stiefbold[1], Thangam Ramar[1], Aditya Agarwal[2], Leo Q. Wan[1,2,3,4,*]

[1]Department of Biomedical Engineering, Rensselaer Polytechnic Institute, Troy, NY 12180, USA

[2]Department of Biological Sciences, Rensselaer Polytechnic Institute, Troy, New York 12180, USA

[3]Center for Biotechnology and Interdisciplinary Sciences, Rensselaer Polytechnic Institute, Troy, NY 12180, USA

[4]Center for Modeling, Simulation, and Imaging in Medicine, Rensselaer Polytechnic Institute, Troy, New York 12180, USA

*Correspondence to:

Leo Q. Wan

Professor of Biomedical Engineering & Biological Sciences

Rensselaer Polytechnic Institute

Biotech 2147, 110 8th Street, Troy, NY 12180, USA

518-276-2505 (Office); 518-276-3035 (Fax); wanq@rpi.edu; https://wan-lab.bme.rpi.edu

A

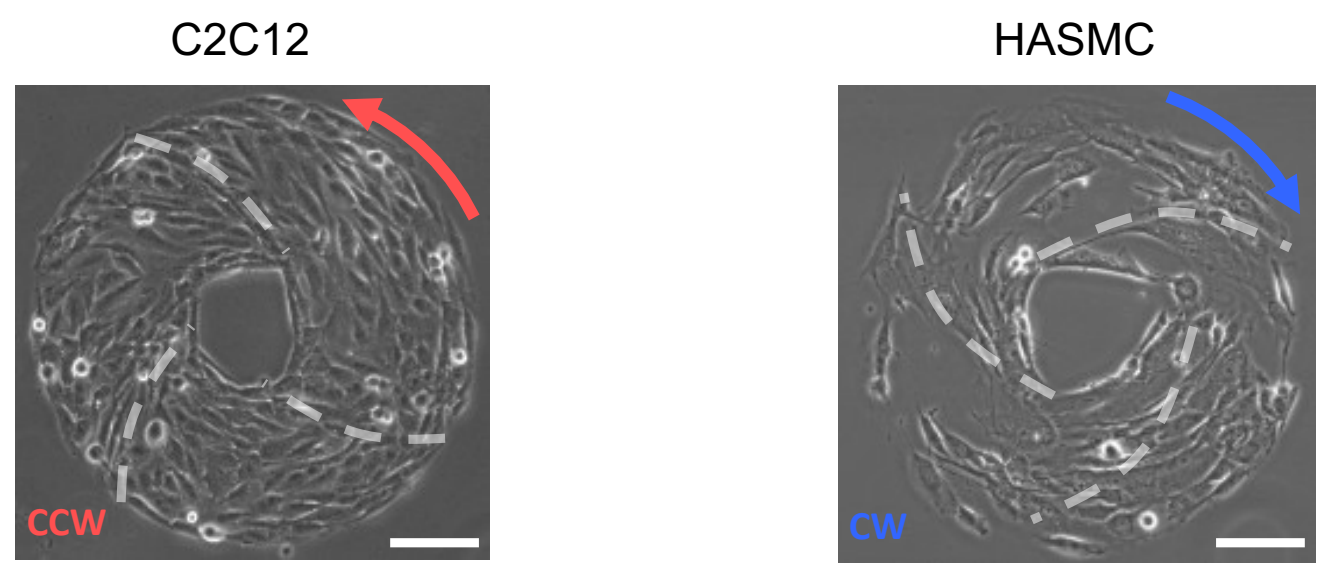

B

	Total	Non-chiral	CW	CCW	Chiral Factor	P-value
C2C12	58	14	1	**43**	0.72	5.12×10^{-12}
HASMC	95	32	**53**	10	-0.45	3.38×10^{-08}

Figure S1. Distinct chiral alignment of C2C12 cells and HASMCs on two-dimensional micropatterns. (**A**) Representative phase-contrast images showing counterclockwise (CCW) alignment of C2C12 cells and clockwise (CW) alignment of human aortic smooth muscle cells (HASMCs) on ring-shaped micropatterns. White dashed lines indicate the approximate orientation of cells. Scale bar = 100 µm. (**B**) Summary of CW, CCW, and non-chiral alignment patterns on ring-shaped micropatterns. A total of n = 58 C2C12 and n = 95 HASMC ring patterns were analyzed. The chiral factor was calculated as (N_CCW − N_CW)/N_total, with positive and negative values indicating CCW and CW bias, respectively. The frequencies of CW and CCW alignment were compared using a two-tailed binomial test under the null hypothesis of equal probability (p = 0.5). The corresponding P values are shown in the table.

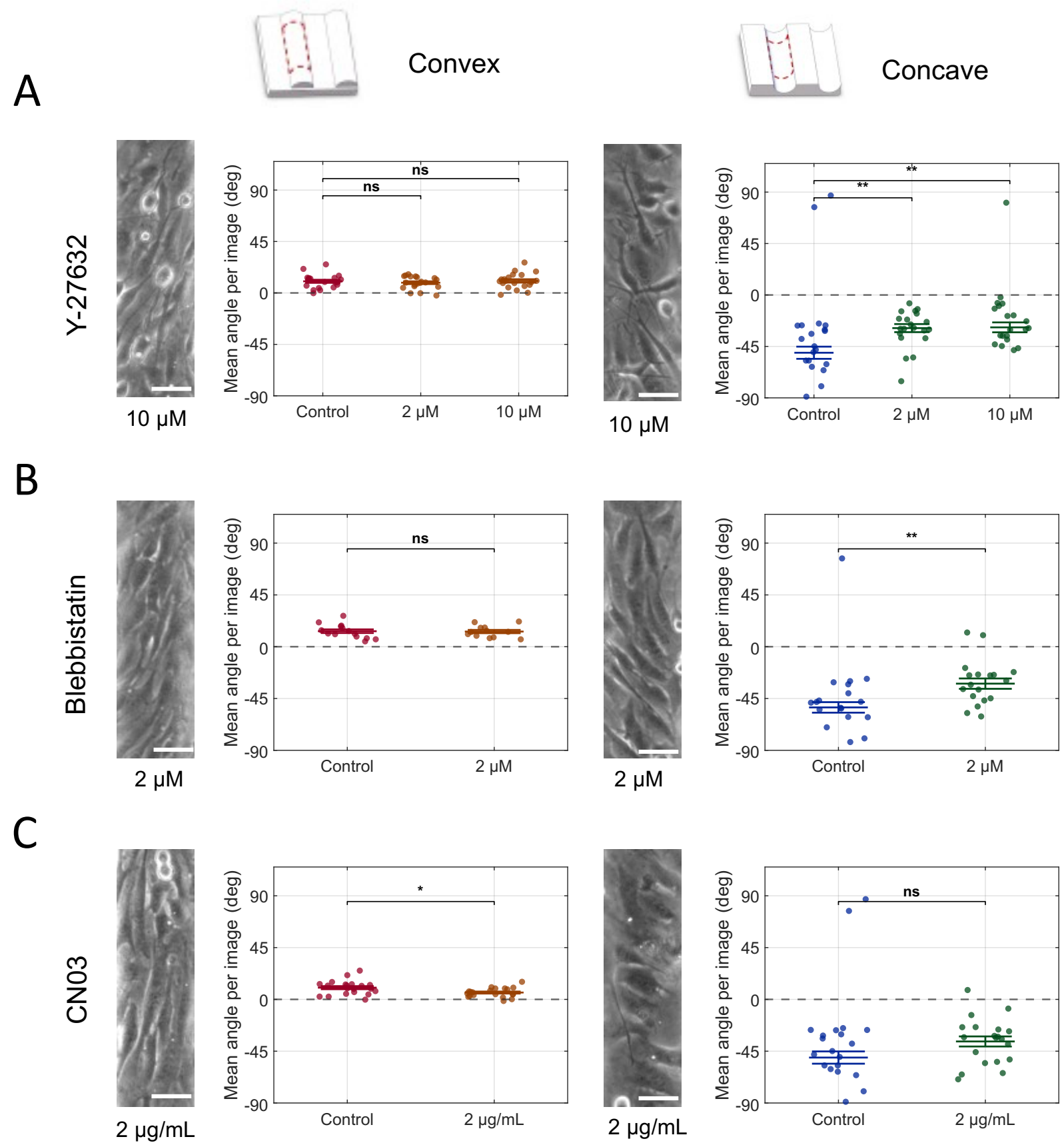

Figure S2. Pharmacological modulation of cellular contractility alters the magnitude but not the direction of cell alignment bias on curved surfaces. (**A**) Representative phase-contrast images and mean orientation angles of C2C12 cells on convex and concave surfaces treated with the ROCK inhibitor Y-27632 (2 or 10 μM). (**B**) Representative phase-contrast images and mean orientation angles of C2C12 cells treated with the myosin II inhibitor blebbistatin (2 μM). (**C**) Representative phase-contrast images and mean orientation angles of C2C12 cells treated with Rho activator II (CN03; 2 μg/mL). All data were collected 24 h after seeding. Each point represents one cropped image; data are shown as mean ± SEM. For (**A**) and (**C**), n = 20 images per condition for both convex and concave surfaces. For (**B**), n = 14 and 12 images for control and blebbistatin-treated cells, respectively, on convex surfaces, and n = 18 images per condition on concave surfaces. Differences in mean orientation between treatment and control groups were assessed using the

Watson–Williams test on doubled angles. For Y-27632 experiments involving multiple pairwise comparisons, P values were adjusted using the Holm method. ns, not significant; *$P < 0.05$; **$P < 0.01$. Scale bars = 50 μm.

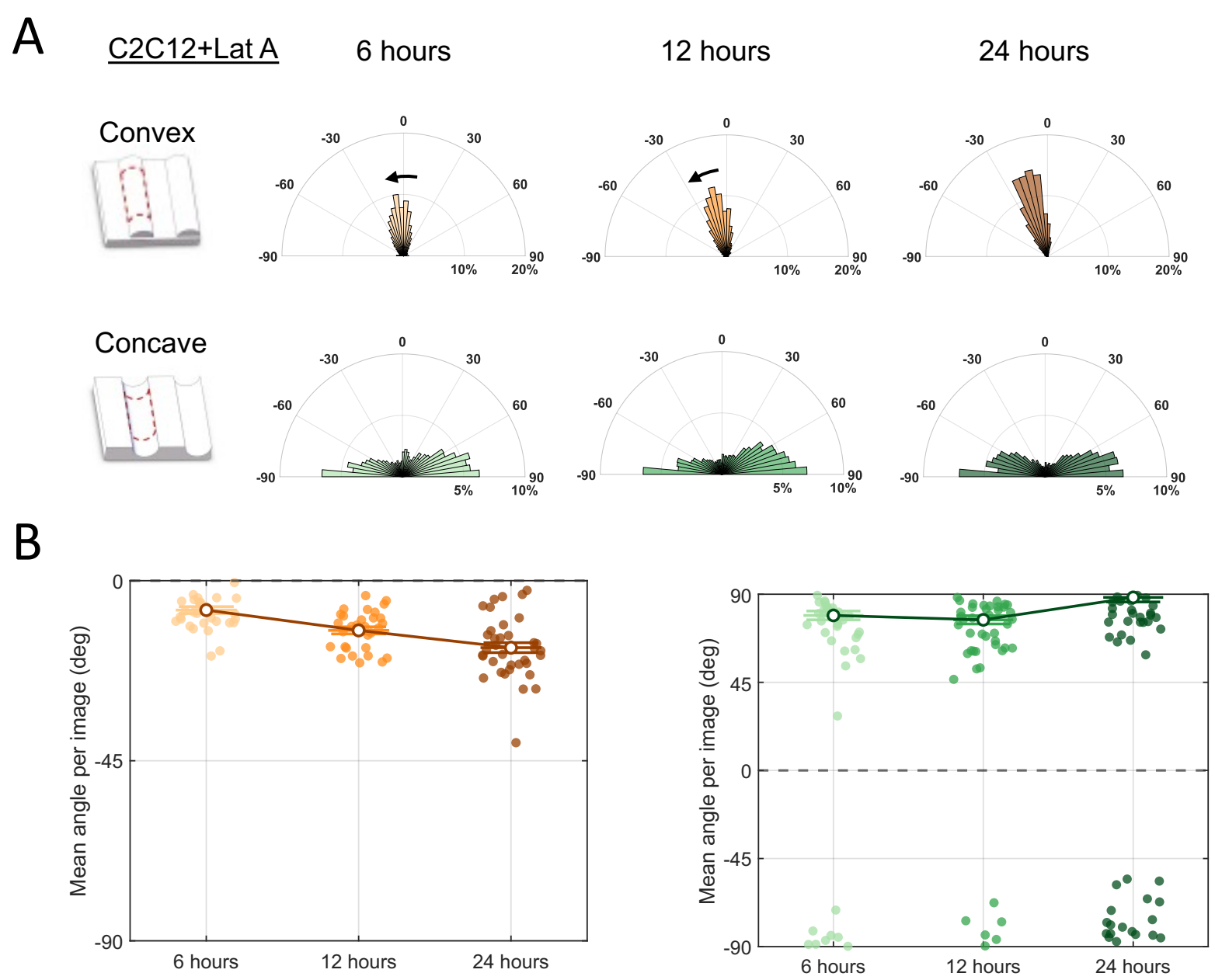

Figure S3. Evolution of the alignment of C2C12 cells on curved surfaces, under the treatment of 50 nM Latrunculin A (Lat A). (**A**) Rose plots showing the distributions of orientation angles of C2C12 cells treated with 50 nM Lat A on convex and concave surfaces at 6, 12, and 24 h after seeding. (**B**) Mean orientation angles of Lat A-treated C2C12 cells on convex (orange) and concave (green) surfaces at 6, 12, and 24 h after seeding. Each point represents one cropped image; data are shown as mean ± SEM. n = 33, 30, and 37 images for the 6, 12, and 24 h time points, respectively, on convex surfaces, and n = 38, 44, and 45 images, respectively, on concave surfaces.

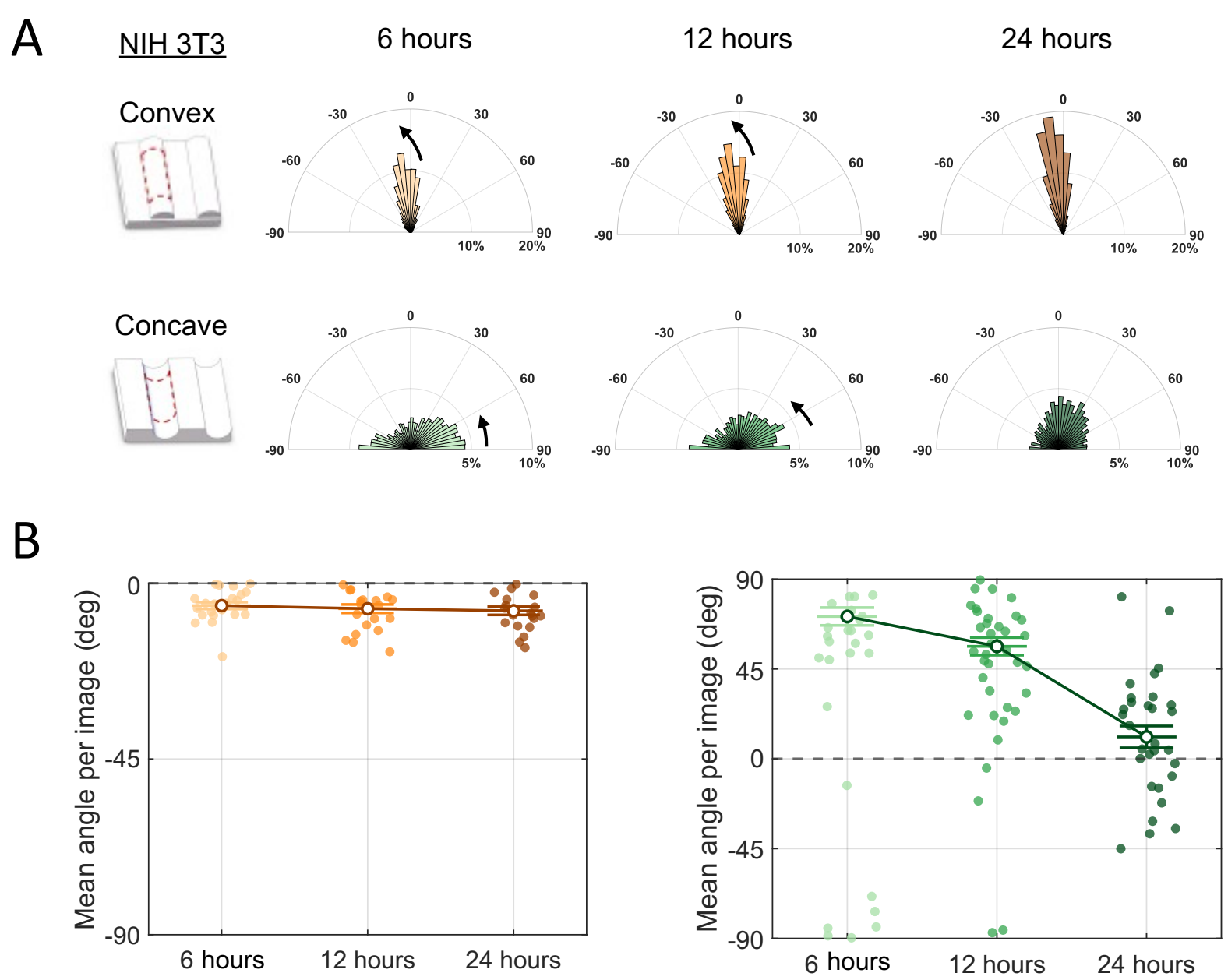

Figure S4. NIH 3T3 cells exhibit a counterclockwise tilting over time on curved surfaces. (**A**) Rose plots showing the distributions of orientation angles of NIH 3T3 cells on convex and concave surfaces at 6, 12, and 24 h after seeding. (**B**) Mean orientation angles of NIH 3T3 cells on convex (left, orange) and concave (right, green) surfaces at 6, 12, and 24 h after seeding. Each point represents one cropped image; data are shown as mean ± SEM. n = 22, 23, and 21 images for the 6, 12, and 24 h time points, respectively, on convex surfaces, and n = 26, 37, and 30 images, respectively, on concave surfaces.

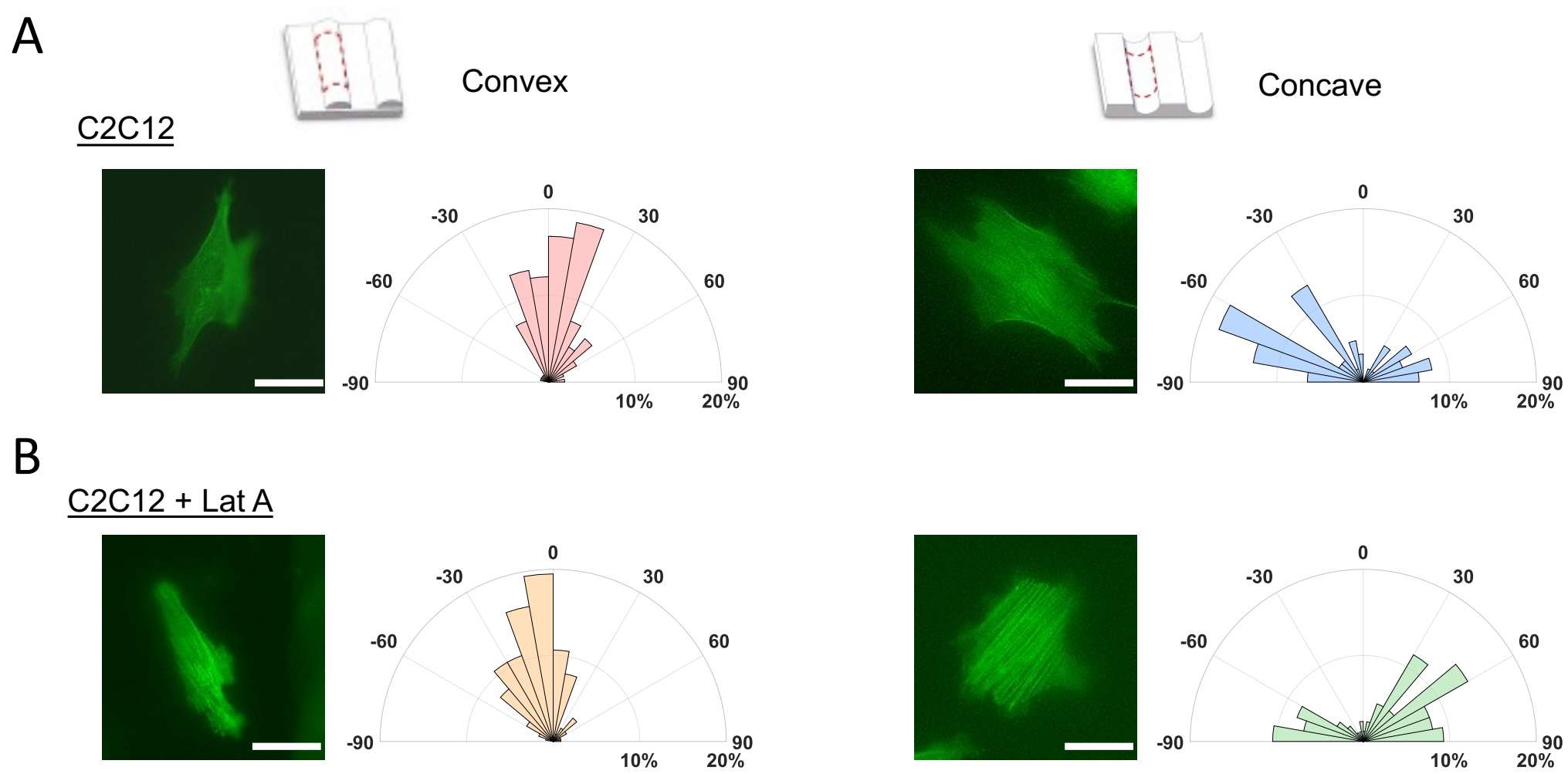

C

	Biased angle (°) on convex surface	Biased angle (°) on concave surface
**C2C12**	7.03 ± 2.65	-73.06 ± 4.97
**C2C12 + Lat A**	-13.52 ± 2.62	71.22 ± 4.00

Figure S5. Curvature-dependent alignment bias emerges at the single-cell level. (**A, B**) Representative fluorescence images and corresponding rose plots of orientation-angle distributions for individual C2C12 cells on convex and concave surfaces at 6 h after seeding, under control conditions (**A**) or treatment with 50 nM Latrunculin A (**B**). F-actin was labeled with phalloidin, and the long axis of each individual cell was used to define the cell elongation direction. Scale bars = 50 µm. (C) Summary of mean orientation angles of individual C2C12 cells under control and Lat A-treated conditions on convex and concave surfaces. Data are presented as mean ± SEM. n = 107 and 62 cells for control C2C12 cells on convex and concave surfaces, respectively, and n = 113 and 86 cells for Lat A-treated C2C12 cells on convex and concave surfaces, respectively.

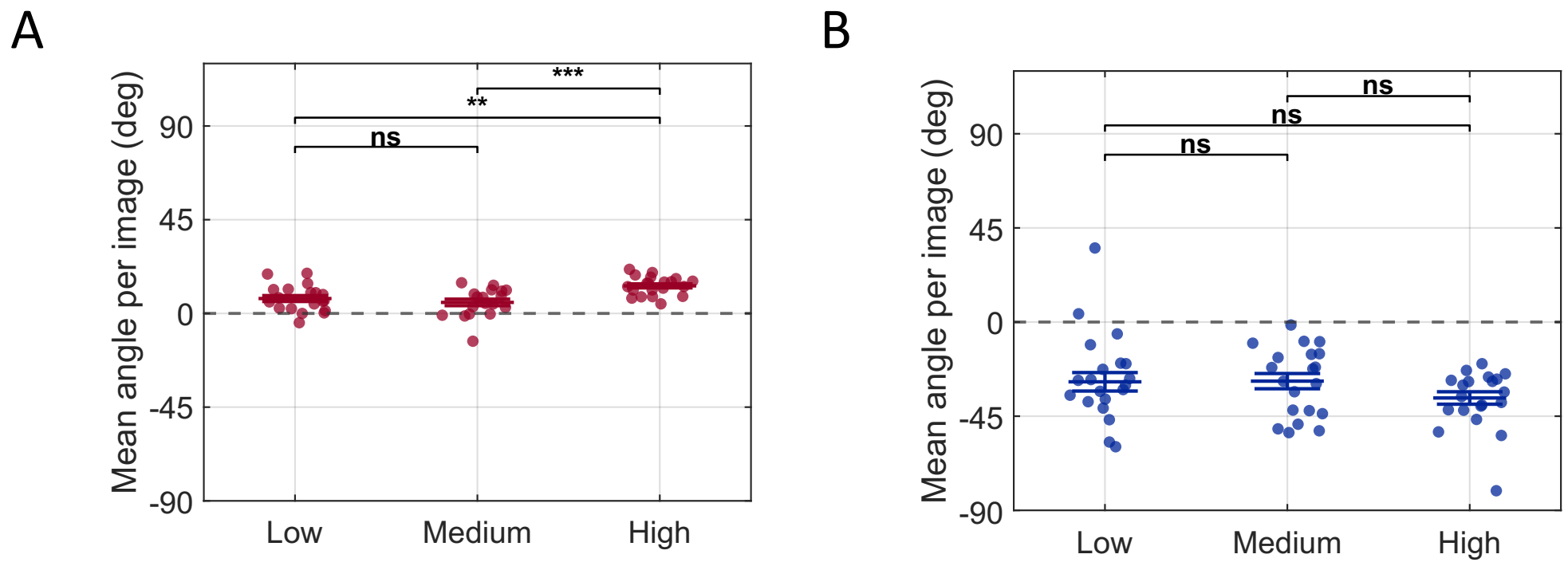

Figure S6. Cell seeding density modulates the magnitude of curvature-dependent alignment bias. (**A**, **B**) Mean orientation angles of C2C12 cells seeded at low (40 k cells/cm^2), medium (80 k cells/cm^2), and high (120 k cells/cm^2) densities on convex (**A**) and concave (**B**) surfaces. Each point represents one cropped image; data are shown as mean ± SEM. n = 19, 18, and 20 images for the low-, medium-, and high-density groups, respectively, on convex surfaces, and n = 20 images per group on concave surfaces. Statistical comparisons were performed using the Watson–Williams test with Holm-adjusted P values. ns, not significant; **$P < 0.01$; ***$P < 0.001$.

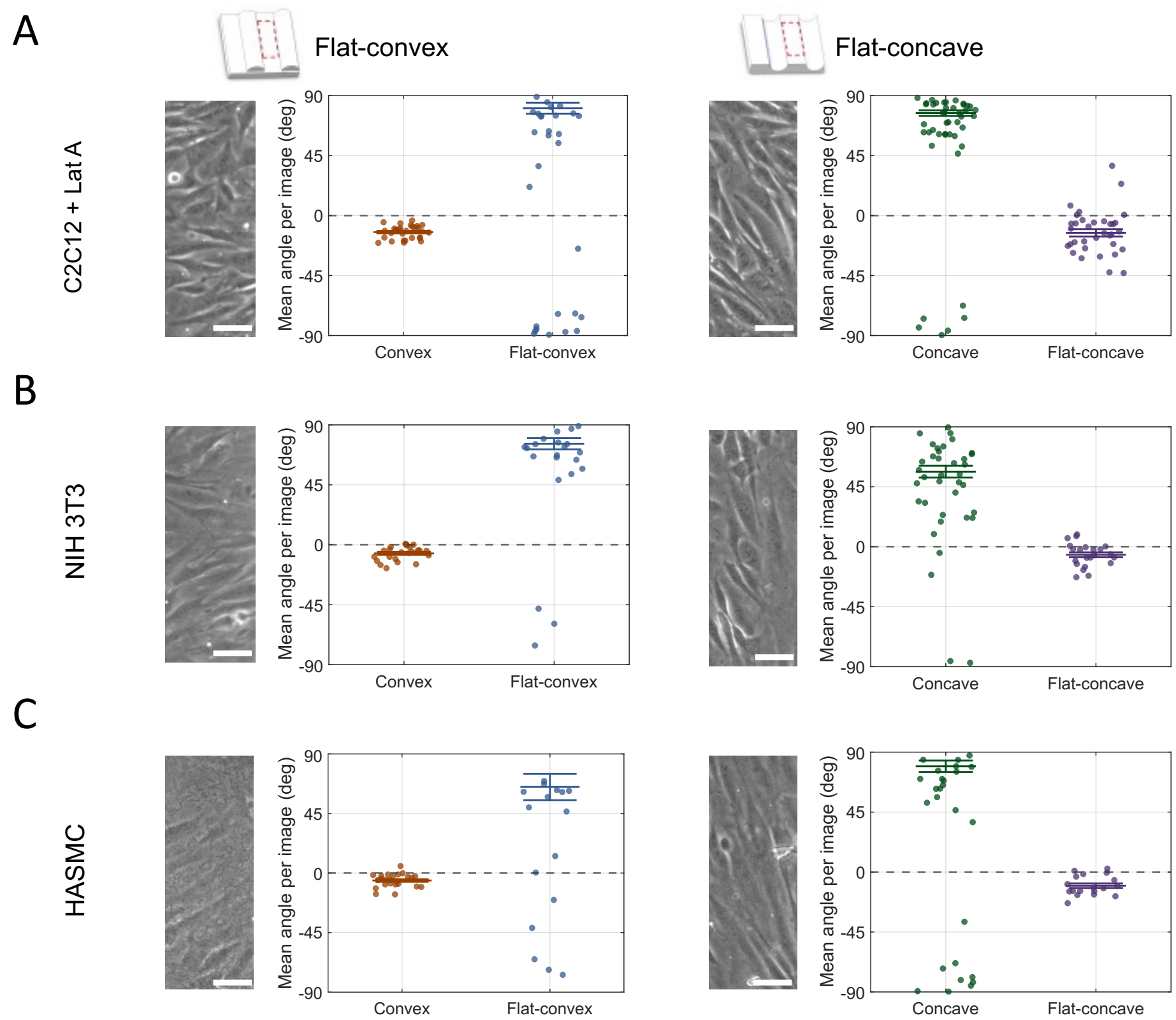

Figure S7. Cells under different conditions exhibit opposite chiral alignment on curved surfaces and adjacent planar regions. (**A**–**C**) Representative phase-contrast images and mean orientation angles of Latrunculin A (Lat A)-treated C2C12 cells (**A**), NIH 3T3 cells (**B**), and human aortic smooth muscle cells (HASMCs) (**C**) on curved surfaces and in the adjacent planar regions. For Lat A-treated C2C12 cells and NIH 3T3 cells, orientation angles are shown at 12 h after seeding, whereas data for HASMCs correspond to 24 h after seeding. Each point represents one cropped image; data are shown as mean ± SEM. Sample sizes for curved and adjacent planar regions, respectively, were n = 30 and 29 for Lat A-treated C2C12 cells, 23 and 21 for NIH 3T3 cells, and 24 and 16 for HASMCs on convex/Flat–convex regions; corresponding sample sizes on concave/Flat–concave regions were n = 44 and 34, 37 and 22, and 26 and 18. Scale bars = 50 µm.

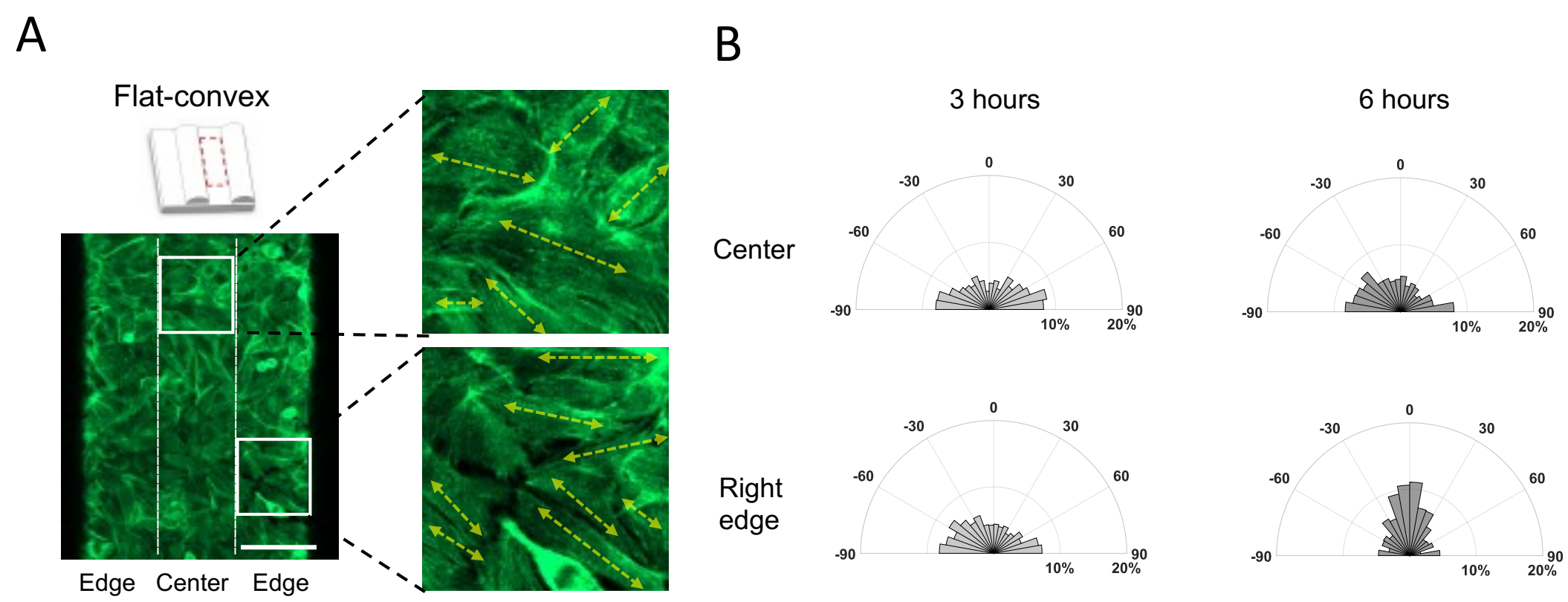

Figure S8. The early alignment of C2C12 cells on flat regions adjacent to the convex surfaces. (**A**) Fluorescence images of C2C12 cells in Flat–convex regions at 3 h after seeding. F-actin was labeled with Alexa Fluor 488-conjugated phalloidin. Magnified views on the right show cells located at the center (top) and edge (bottom) of the planar region. Scale bars = 100 µm. (**B**) Rose plots showing the distributions of cell orientation angles in the center and edge subregions of Flat–convex areas at 3 and 6 h after seeding.

Supplementary Video 1: 24-hour time-lapse imaging of C2C12 cells on convex surfaces. Phase contrast images were acquired every 12 minutes starting 3 hours after seeding. Scale bar = 100 µm.

Supplementary Video 2: 24-hour time-lapse imaging of C2C12 cells on concave surfaces. Phase contrast images were acquired every 12 minutes starting 3 hours after seeding. Scale bar = 100 µm.

Supplementary Video 3: Nuclear tracking of C2C12 cells on convex surfaces. Cell nuclei were stained with Hoechst 33342 and imaged every 20 minutes. Yellow circles indicate detected nuclei, and colored solid lines represent nuclear trajectories.

Supplementary Video 4: Nuclear tracking of C2C12 cells on concave surfaces. Cell nuclei were stained with Hoechst 33342 and imaged every 20 minutes. Yellow circles indicate detected nuclei, and colored solid lines represent nuclear trajectories.

Supplementary Video 5: 24-hour time-lapse imaging of C2C12 cells on Flat-convex regions. Phase contrast images were acquired every 12 minutes starting 3 h after seeding. Scale bar = 100 µm.

Supplementary Video 6: 24-hour time-lapse imaging of C2C12 cells on Flat-concave regions. Phase contrast images were acquired every 12 minutes starting 3 h after seeding. Scale bar = 100 µm.
